# The influence of the position of the laryngeal endoscope laryngeal on videokymography

**DOI:** 10.1016/S1808-8694(15)31252-0

**Published:** 2015-10-20

**Authors:** Paulo Augusto de Lima Pontes, Glaucya Madazio, Mara Behlau, Luiz Alian Cantoni

**Affiliations:** Ph.D. in Medicine, Escola Paulista de Medicina, 1970. Post-doctorate in Medicine, Escola Paulista de Medicina, 1981. Full Professor, Discipline of Otorhinolaryngology, Department of Ophthalmology/Otorhinolaryngology, Escola Paulista de Medicina, 1989. Full Professor, Department of Otorhinolaryngology and Human Communication Disorders, Escola Paulista de Medicina, 1991. Full Professor of Otorhinolaryngology, UNIFESP-EPM and Coordinator of the Program of Post-graduation in Otorhinolaryngology and Head and Neck Surgery, Federal University of Sao Paulo; Speech and Hearing Therapist, Master in Human Communication Sciences - Unifesp. Specialist in Voice, CFFa, clinical speech therapist, Professor of the Course of Specialization in Voice, CEV; Specialist in Voice, Master and Ph.R. in Human Communication Disorders. Director of Centro de Estudos da Voz de Sao Paulo and Coordinator of the Specialization Course, CEV; Master in Sciences, Program of Post-graduation in Otorhinolaryngology and Head and Neck Surgery, Federal University of Sao Paulo - Escola Paulista de Medicina - UNIFESP - EPM. Otorhinolaryngologist. Study performed at INLAR - Instituto da Laringe

**Keywords:** larynx, voice, vocal cords

## Abstract

Videokymography is a new high-speed imaging technique to investigate vocal fold vibration. The system has been considered useful in the analysis of irregular signals, making it possible to observe left-right asymmetries, open quotient, propagation of mucosal waves, and movement of the upper and lower margins. The aim of the present study was to verify the correspondence of the videokymographic images with larynx exposition angle variation. Three Brazilian adult female subjects, with no vocal complains, were evaluated. Videokymographic images were obtained with the laryngeal endoscope 90o positioned on the measuring line, perpendicular to the glottal axis - zero degree, during a sustained “ae” vowel, using habitual frequency and intensity. The procedure was repeated twice and adjusted by rotating the camera in fifteen degrees from the perpendicular line to the right and to the left. The results showed clear differences depending on the position of the endoscope, suggesting the standardization of laryngeal exposition to interpretate videokymographic images correctly.

## INTRODUCTION

Vocal quality is determined by the mucosa vibration of the vocal folds. The quantification of the mucosa vibration is an important task that is difficult to be performed.

Modern laryngoscopic exams are efficient in the assessment of vocal behavior and visualization of vocal folds, in addition to allowing coupling of a video camera, used to record images. Because the video camera can record only 25 to 30 images per second, and both male and female vocal frequency exceeds this value, it is impossible to assess these vibrations in slow motion. To that end, some additional techniques are required to have images that enable the assessment of the real vibration movement of the vocal folds.

The clinical practice in voice is normally faced by irregularities and instability of vocal fold vibration in dysphonic voices, which hinders the formation of stroboscopic image. Videokymography is an exam capable of overcoming this disadvantage, because it is theoretically capable of recording these irregular vibrations.

Videokymography was developed by Dutch researchers^1^, and it is considered one of the most recent technological advances in laryngeal semiology. The technique was developed to facilitate the assessment of vocal folds, providing more accuracy and objectivity in the results, especially when stroboscopy failed2., 3., 4..

Videokymography used digital technology for ultra fast analysis of vibration and even though it has been developed for the study of the vocal folds, it may be useful in other vibration systems.

To that end, a system of special CCD video - Charge Couple Device was developed by Voice Research Laboratory (University of Groningen, Nederland) and Lambert Instruments BV (Nederland), in which a camera, named videokymographer is capable of working in two different ways. First, the standard mode, in which the system works as a normal commercial camera, capturing 25 to 30 frames per second, depending on the system (CCIR or NTSC, respectively). In the second mode (high speed), the videokymographer is capable of capturing 8,000 frames per second and transmit them to a monitor^1^.

The principle of the videokymography is that each frame in NTSC system is comprised by 525 horizontal lines. These lines are read point to point, and successively, starting from the left upper side and finishing on the right lower point of the frame. Then, CCD camera system reads these 525 lines in two groups of 262.5 lines each, alternatively. Thus, the first line of the first group is followed by the first line of the second group, and then the second line of the second group, and so on. These groups of lines are known as field A and field B. Videokymography discards field B and reading is made in each frame, only in field A. However, rather than reading all 262.5 lines in the whole field A, the system reads only one of these lines and then it is read 262.5 times in thirty parts of a second, which represents approximately 7,812.5 readings of the same line in one second.

The reading made by the videokymography is transmitted to the commercial TV monitor in each frame, these 7,812.5 readings are presented in only 144 horizontal lines that will occupy a period of time of 18.4 milliseconds.

Given that vibration of vocal folds occur in a rage of distribution of 70 to 250 cycles per second in the modal record, and having the possibility to perform in the same period 7,812.5 readings, it means that vibration of vocal folds may be really seen in slow motion.

However, it is important to point out that the image presented is the reference of one single line in the image captured, even though it comprises the whole image of the presentation screen. With this single line, it will be possible to analyze only a narrow horizontal segment of laryngeal image.

Owing to the fact that color is not essential to the recognition of images, videokymographer for simplification reasons, works in black and white, that is, in variations of gray.

Videokymography uniquely assesses all types of irregularities of vibration, regardless of vocal quality and level of abnormality; it includes visualization of open and closed phase of glottic cycle, it identifies small left-right asymmetries, differences in the opening quotient of vocal folds, lateral propagation of mucosa wave and movement of upper and lower margins of vocal folds mucosa^1^^,^^2^^,^^5^^,^^6^. Thus, videokymography is an additional diagnostic resource, providing a very accurate analysis of the pathophysiology of vocal disorders^4^^,^^7^.

Videokymographic exams performed in patients without vocal complaints present great variability in vibration pattern of vocal folds^6^. Some resulting asymmetries were not identified by stroboscopy, but were easily identified by videokymography. These asymmetries may occur at the glottic level or because of problems in the technical procedure, with incorrect introduction of telescope. If these asymmetries do not take place at the glottic level, the diagnosis of asymmetric vibration may lead to false positive results.

Some factors related to variability of vocal fold vibration pattern were specified^6^: the positioning of the telescope, both in relation to the point to be studied, considering that vibration is different in the vocal folds, and in relation to rotation of telescope in relation to the midline, because it is not in perpendicular position - zero degree, which may generate an illusion image; vocal quality, in which small affections to frequency and intensity interfere in vibration of vocal folds.

In a study performed with dog larynges^8^, there was linear relation of amplitude of mucosa wave excursion, frequency and subglottic pressure. When larynges are elongated, the amplitude of the mid third of vocal folds is reduced, whereas frequency increases with subglottic pressure. In this study, we did not observe direct correlation between subglottic pressure and difference in the movement phase of mucosa wave.

Two careful measurements should be taken during videokymography assessment: the first one refers to the fact that images are obtained in different points of the vocal folds owing to the existing differences in vibration pattern in all their extension; the second, the position of the telescope during the images, which should remain always in the selected line^3^. The impossibility to visually control the selected cordal region during the performance of videokymography is a limitation of the technique. Another limiting factor is the fact that the selected line should correspond to the first line on the monitor screen, such as scheduled by most videokymographers, and it is not always possible to obtain the videokymographic image of the desired cord region, because upon mobilizing the telescope to bring the first line of the screen to the desired region, some factors, such as opening of epiglottis or posterior position of the tongue may prevent the visualization of the selected region.

As a routine, there is no concern in maintaining the laryngeal telescope in a fixed position for the assessment of the larynx, with or without stroboscopy. The examiner searches for the best laryngeal image possible to be collected, for further diagnostic precision.

## OBJECTIVE

The purpose of the present study was to check the correspondence of images obtained by videokymography with variation of angle of exposure of the larynx in relation to the telescope in three female subjects without vocal complaints.

## METHOD

Three adult female subjects participated in the study, aged 25 to 30 years, without vocal complaints and no laryngeal abnormalities at telelaryngoscopy. All subjects were assessed at Instituto da Laringe - INLAR, in Sao Paulo.

The technique of videokymography is similar to the classical procedure of laryngoscopy, with the subject at seated position, open mouth, protracted tongue involved in gauze and maintained in position by light digital pulling by the Otorhinolaryngologist. After spraying xylocaine at 2% for topical anesthesia of oropharynx, the rigid laryngoscope was introduced through the mouth of the subject up to the pharynx through the medial plan, position named zero, that is, with the vision line perpendicular to the horizontal plan that passes through the glottis.

To perform the study, when we reached food visibility of the whole extension of the vocal folds, in standard mode, the line that corresponded to the central portion of the mid third of the membranous portion of the vocal folds was selected and the videokymography pedal was activated. The videokymography used was KAY ELEMETRICS INC., model 8900, coupled to telescope MACHIDA® of 90°. The first horizontal line of the monitor coincided with the central portion of the glottis and was assessed by videokymography. The videokymographic images were recorded during the emission of sustained vowel /ae/, at habitual frequency and intensity.

Next, the procedure was repeated but we displaced the laryngoscope to the right side of the patient, turning it anti-clockwise to the examiner, around its own axis, maintaining the glottic region centered in the monitor and correcting the presentation with rotation of the camera in clockwise direction. When the rotation reached 15 degrees, the displacement was interrupted and videokymography was recorded. The same procedure was performed with displacement to the left (rotation of laryngoscope in clockwise direction). The angulation of the telescope was measured by the rotation of the camera using a transferring device.

The vocal frequency was monitored using an electronic keyboard YAMAHA PSR 140 and intensity was monitored through sound pressure meter MINIPA MSL-1350, placed at a distance of 30cm from the subjects’ mouth. We monitored constantly the frequency and intensity so that if there were variations in images they would only be due to changes in position of telescope and not resulting from these variables.

Images were recorded in videocassette and later they were analyzed and reproduced frame by frame. The criteria used for selecting the frames were clear tracing in the central portion of the period of production, with image defined by the upper and lower margins of the mucosa wave, with beginning and ending of opening and closing of the vocal folds during the opening phase of the vibration cycle.

**Figure 1:**
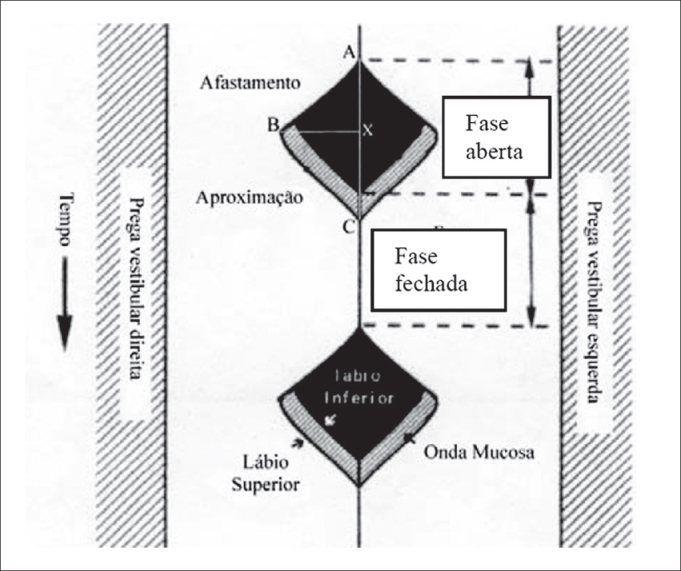
Schematic representation of a vibration cycle analyzed by videokymography and the assessed parameters.

The schematic representation is presented in Figure 1 to better understand the videokymographic images and the parameters assessed.

Once images were selected, we made photographic printing of each production, amounting to 9 pictures. The photos were copied by SNAP SCAN 1212 AGFA and processed in a microcomputer MACINTOSH Power Computing - Power Center, in software Adobe Premiere.

In each photo we drew a vertical line corresponding to the limits between the two vocal folds (time axis), and two horizontal lines, passing through the mucosa wave of each vocal fold (distance axis). With those 3 lines, the following points were marked:

A -point of the vertical line that corresponds to the beginning of the opening of the open phase;
B -point of the horizontal line that corresponds to the beginning of the closing stage of the open phase, in each vocal fold (Bd and Be);
C -point of the vertical line that corresponds to the open phase; Points X, to wit:
Xd -crossing point of vertical line with horizontal line of the right vocal fold;
Xe -crossing point of the vertical line with the horizontal line of the left vocal fold.

In vibration cycles with similar times of opening periods, points Xd and Xe coincide.

Based on these points, we considered the following parameters:

1. Opening time of each vocal fold - distance between points A and the respective X (Xd and Xe);
2. Closing time of each vocal fold - distance between the respective points X and point C;
3. Maximum opening of each vocal fold - distance between the respective points X and point B.

Measurements to both vertical and horizontal axis were made in pixels.

Using these measurements, we made the comparisons between vocal folds in the three different positions of the telescope.

## RESULTS

The first published texts about videokymography were not concerned with specific details about correct introduction of the telescope1., 2., 3., 4., 5.. One of the most recent publications 6 warned about the position of the telescope during the recording of videokymographic images, when they studied the variability of vocal fold vibration pattern with videokymography.

The positioning of the telescope and the point to be studied were related 6 considering that the vibration is different along the vocal folds, and the rotation of the telescope from the midline, because if it is not at a perpendicular position - zero degree, could generate an asymmetrical image that is simply an illusion.

Thus, through the study of three female subjects without vocal complaints we managed to have the correspondence of images by videokymography with variation of the angle of laryngeal exposure in relation to the telescope that is rotated.

Table 1 presents the time of opening and closing of the vocal folds in the open phase measured in pixels, in addition to maximum opening of the vocal folds in the three subjects with the telescope positioned at the midline - zero degree. Only the laryngeal image of subject three presented symmetrical opening and closing time between the right and vocal folds.

**Table 1:** Opening and closing times and closing of vocal folds and the respective maximum opening times measured in pixels, with the telescope positioned at zero degree.

Subject		Opening Time			Closing Time	Maximum Opening
	R	L	R	L	R	L
1	0,73	1,12	0,99	0,64	0,65	0,64
2	1,80	1,36	1,32	1,76	1,06	1,20
3	0,79	0,79	0,75	0,75	0,69	0,76

**Graph 2:**
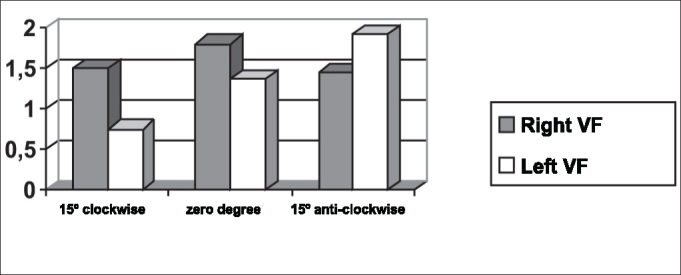
Opening time of vocal folds measured in pixels, with the telescope introduced at three different rotations in subject 2.

**Graph 3:**
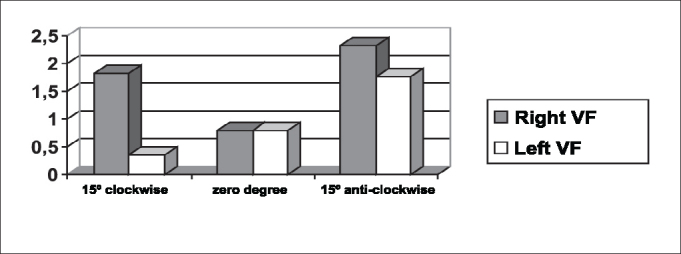
Opening time of vocal folds measured in pixels, with the telescope introduced at three different rotations in subject 1.

**Figure 2:**
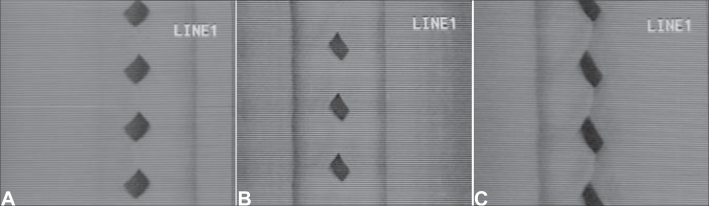
Videokymographic images of subject 1 producing sustained vowel /ae/ in modal register and fundamental frequency of 261Hz, vocal intensity of 83dB. 2a. laryngeal telescope rotated at 15 o clockwise; 2b. laryngeal telescope positioned at midline - zero degree; 2c. telescope rotated at 15 degrees, anti-clockwise.

**Figure 3:**
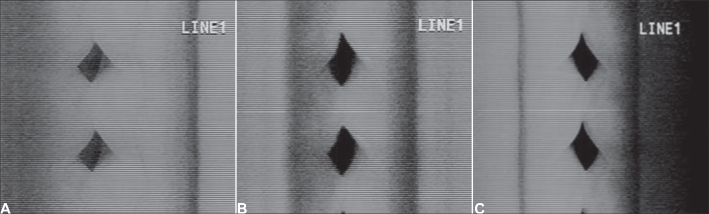
Videokymographic images of subject 2 producing sustained vowel /ae/ in modal register and fundamental frequency of 174Hz, vocal intensity of 80dB. 3a. laryngeal telescope rotated at 15 o clockwise; 3b. laryngeal telescope positioned at midline - zero degree; 3c. telescope rotated at 15 degrees, anti-clockwise.

**Figure 4:**
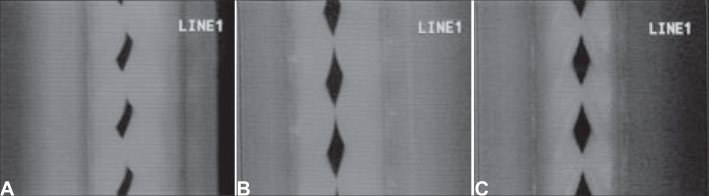
Videokymographic images of subject 3 producing sustained vowel /ae/ in modal register and fundamental frequency of 208Hz ands vocal intensity of 74dB. 4a. laryngeal telescope rotated at 15 o clockwise; 4b. laryngeal telescope positioned at midline - zero degree; 4c. telescope rotated at 15 degrees, anti-clockwise.

The symmetry of opening and closing in the open phase of the vibration cycle was checked in Graph 2, Graph 3, which showed opening and closing time of subject 3, respectively. In Figure 4b we can see the videokymographic image of subject 3 with symmetry of opening and closing times, which was even more evident when compared to the other two subjects presented in Figure 2, Figure 3, all of them with the telescope positioned at zero degree.

In table 1, we can also observe that only the laryngeal image of one of the subjects - subject 1 - presented symmetry in maximum opening of vocal folds, that is, lateralization of right and left folds, even though there had not been symmetry in opening and closing times during the open phase. Graph 7 evidenced this symmetry, observed also in Figure 2b.

**Graph 6:**
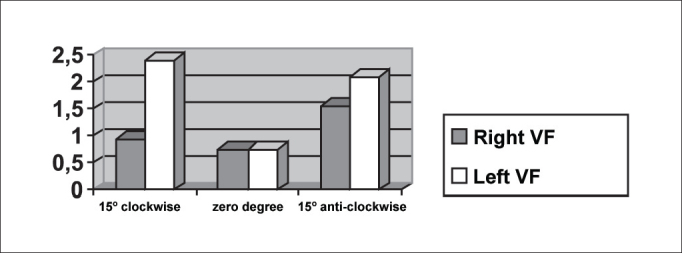
Closing time of vocal folds measured in pixels, with the telescope introduced at three different rotations in subject 3.

Despite the symmetries found in subjects without vocal complaints, such as in subjects 1 and 2 (Table 1), it is interesting to study and see what is the real clinical implication of this findings, which is capable of causing laryngeal imbalance when there is, for example, increase in vocal demand.

The same subjects were assessed, according to Table 2, with the telescope rotated to $15\underline{{}^{\circ}}$ clockwise. Subject 3, with symmetrical image when the telescope was positioned at the midline, did not maintain the same condition with the rotation. This fact can be evidenced with the comparison of Figures 4a and 4b, and also by observing Graph 2, Graph 3.

**Table 2:** Opening and closing times and closing of vocal folds and the respective maximum opening times measured in pixels, with the telescope rotated at $15\underline{{}^{\circ}}$ clockwise.

Subject		Opening Time			Closing Time	Maximum Opening
	R	L	R	L	R	L
1	0,95	0,85	0,99	0,64	0,95	0,89
2	1,49	0,75	0,98	1,72	1,00	0,75
3	1,83	0,36	0,94	2,41	0,48	0,41

**Graph 1:**
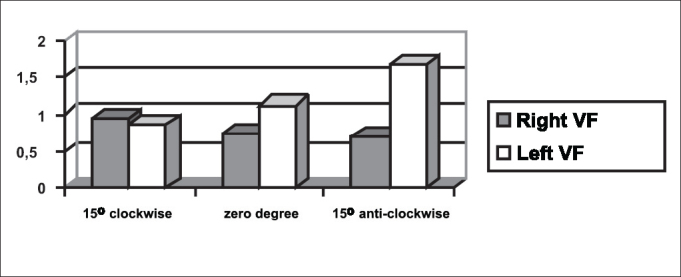
Opening time of vocal folds measured in pixels, with the telescope introduced at three different rotations in subject 1.

**Graph 4:**
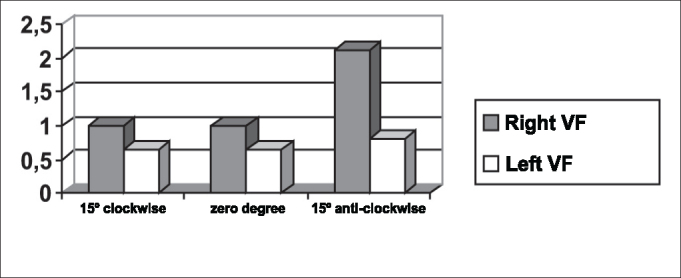
Closing time of vocal folds measured in pixels, with the telescope introduced at three different rotations in subject 1.

Conversely, subject one showed in her laryngeal image a small difference in time of opening of right and left folds, making it almost symmetrical, as shown in Table 2 and Graph 1. However, times referring to closing were the same, regardless of the telescope position, observed in Table 2 and Graph 4. The comparison of Figures 2a and 2b evidenced affections to opening times of vocal folds, making image 2a almost symmetrical.

**Graph 7:**
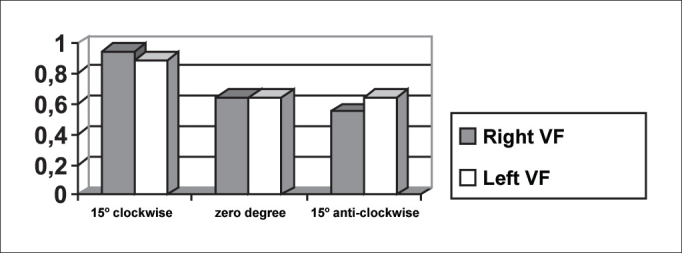
Maximum closing time of vocal folds measured in pixels, with the telescope introduced at three different rotations in subject 1.

**Graph 8:**
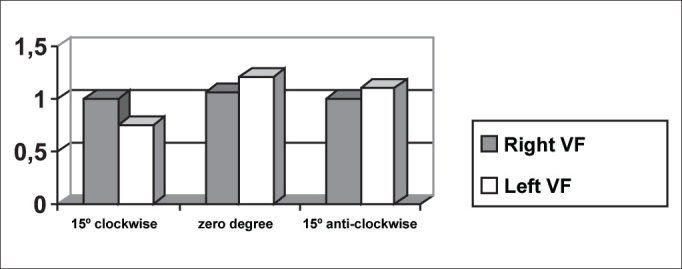
Maximum closing time of vocal folds measured in pixels, with the telescope introduced at three different rotations in subject 2.

As to maximum opening of vocal folds, we can observe in Table 2 that all subjects presented left-right asymmetry in laryngeal images, confirmed by Graph 6, Graph 7, Graph 8, which represented the maximum opening time in the three subjects.

In Table 3, the procedure was performed with the telescope turned at $15\underline{{}^{\circ}}$ anti-clockwise. We could see that the three subjects presented asymmetrical values, both for closing and opening times, shown in Graphs1., 2., 3., 4., 5., 6.. Conversely, as to maximum opening, the values were practically symmetrical for the three tested subjects, as shown in Graph 6, Graph 7, Graph 8.

**Table 3:** Opening and closing times and closing of vocal folds and the respective maximum opening times measured in pixels, with the telescope rotated at $15\underline{{}^{\circ}}$ anti-clockwise.

Subject		Opening Time			Closing Time	Maximum Opening
	R	L	R	L	R	L
1	0,69	1,65	2,10	0,80	0,55	0,65
2	1,45	1,92	1,77	1,30	1,00	1,10
3	2,32	1,77	1,55	2,10	0,58	0,60

Table 4 compared times of opening of vocal folds during the open phase in the three studied positions of introduction of laryngeal telescope. Subject one presented asymmetrical image with the telescope positioned at midline - zero degree. With $15\underline{{}^{\circ}}$ rotation clockwise, the asymmetry was mild, as if a small rotation had corrected the first image. When the telescope was rotated to $15\underline{{}^{\circ}}$ anti-clockwise, the asymmetry was even greater. Figure 2, presented in documentation, shows evidence of this difference and Figure 2a was obtained with the telescope rotated $15\underline{{}^{\circ}}$ clockwise, Figure 2b with the telescope positioned at midline - zero degree, and Figure 2c with the telescope rotated at $15\underline{{}^{\circ}}$ anti-clockwise.

**Table 4:** Opening time of vocal folds measured in pixels, with the telescope introduced at three different rotations, measured in degrees.

Subject				Opening time		
		$15\underline{{}^{\circ}}$ clockwise			Zero degree	$15\underline{{}^{\circ}}$ anti-clockwise
	D	E	D	E	D	E
1	0,95	0,85	0,73	1,12	0,69	1,65
2	1,49	0,75	1,80	1,36	1,45	1,92
3	1,83	0,36	0,79	0,79	2,32	1,77

Table 5 showed values of closing of vocal folds during the opening phase in the three studied positions of laryngeal telescope introduction. It is interesting to notice that in subject one we observed the same laryngeal behavior in two different positions, that is, both for the telescope positioned in the midline and the $15\underline{{}^{\circ}}$ rotation clockwise. With the telescope rotated $15\underline{{}^{\circ}}$ anti-clockwise, the pattern changed. Figure 2 shows the repetitive pattern of closing time, both in Figure 2a and 2b, different from Figure 2c. The same result is shown in Graph 4, which represents the closing time found in subject one, in the three different positions of introduction of the telescope.

**Table 5:** Closing time of vocal folds measured in pixels, with the telescope introduced at three different rotations, measured in degrees.

Subject			Closing			
		$15\underline{{}^{\circ}}$ clockwise			Zero degree	$15\underline{{}^{\circ}}$ anti-clockwise
	R	L	R	L	R	L
1	0,99	0,64	0,99	0,64	2,10	0,80
2	0,98	1,72	1,32	1,76	1,77	1,30
3	0,94	2,41	0,75	0,75	1,55	2,10

**Graph 5:**
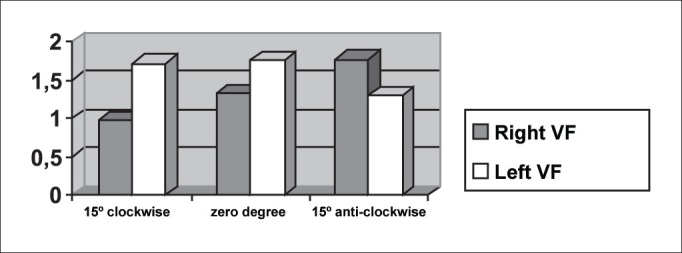
Closing time of vocal folds measured in pixels, with the telescope introduced at three different rotations in subject 2.

As we could see in Graph 5 and Figure 3, the closing times of subject two varied in the three different telescope positions. With the $15\underline{{}^{\circ}}$ rotation clockwise the closing time of right vocal fold was shorter than the closing time of left fold. With the telescope at midline, the difference between the times was reduced, despite the fact that the right vocal fold presented shorter time, and with $15\underline{{}^{\circ}}$ rotation anti-clockwise the closing time of the vocal folds was inverted, that is, the right vocal fold presented closing time longer than the left vocal fold, as if the images had been inverted when the telescope was rotated $15\underline{{}^{\circ}}$ clockwise.

In Table 5 we can see that the laryngeal image of subject 3 presented symmetry of time of closing of right and left vocal folds with the telescope positioned at zero degree. With rotation of telescope, in both directions, the symmetry was not maintained, as expected, but it presented an inverted image, as we can see in Graph 6.

Table 6 pointed to the values of maximum opening of vocal folds, or the maximum lateralization reached by the vocal folds in the three studied subjects. It is important to highlight that the behavior of this parameter was very similar in the three subjects, regardless of the position of the telescope, especially in subjects 1 and 3. That is, the times did not vary much with the modifications to the introduction of laryngeal telescope, easily identified in Graph 6, Graph 7, Graph 8.

**Graph 9:**
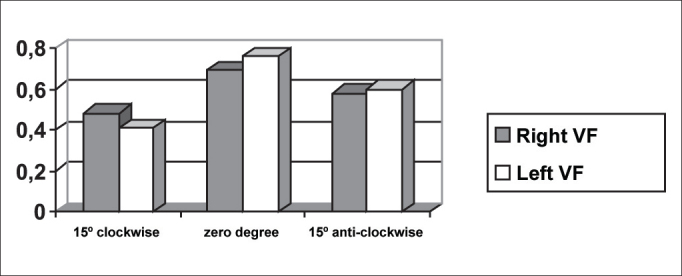
Maximum closing time of vocal folds measured in pixels, with the telescope introduced at three different rotations in subject 3.

**Table 6:** Maximum opening of vocal fold measured in pixels, with telescope introduced at three different rotations, measured in degrees.

Subject			Maximum Opening			
		$15\underline{{}^{\circ}}$ clockwise			Zero degree	$15\underline{{}^{\circ}}$ anti-clockwise
	R	L	R	L	R	L
1	0,95	0,89	0,65	0,64	0,55	0,65
2	1,00	0,75	1,06	1,20	1,00	1,10
3	0,48	0,41	0,69	0,76	0,58	0,60

As to opening time, according to Figure 1, Graph 2, all studied subjects showed an inversion of image to the right and to the left, depending on the rotation direction of the telescope.

Graph 3, Graph 5, which represent the time of closing of vocal folds in the three subjects, did not show this image inversion depending on introduction of the telescope. The behavior of closing time was different in the three studied subjects.

The data presented showed that the difficulties in performing the videokymography technique should be overcome, given that minor adjustments to angulation of laryngeal exposure are enough to modify the results.

Despite the literature3., 4., 5., 6., videokymography is considered an excellent exam to assess dysphonic voices; initially, videokymographic images of adapted voice should be studied and interpreted so that the procedure can be used for dysphonic voices as well.

Only one study was performed with dysphonic voice^4^ and concluded that videokymography is a useful instrument in daily vocal practice, provided that it is associated with other procedures, such as stroboscopic assessment, auditory perceptual assessment of vocal quality and acoustic analysis.

Currently, the laryngological routine involves stroboscopy as the main diagnostic procedure for laryngeal affections. To perform the exam with more precision, the clinician should use some strategies that many times modify the position of the endoscope to improve the view, be it to reinforce aspects of the areas to be analyzed or to favor the focus and the light in the exam. In videokymography this strategy is not recommended and should be avoided. First of all, given that videokymography assesses in real time only one point of the vocal folds, it implies a controlled and careful procedure from a technical perspective. Secondly, we can observe that small modifications in the rotation of the telescope are enough to significantly modify the diagnosis.

It has been suggested^7^ that color videokymography could be performed to facilitate the recognition of structures and vibration phenomena. However, technical improvement should be primarily related with some maneuvers to support the clinician to maintain exactly the same position of the telescope during the videokymography procedure.

One of the disadvantages of videokymography is the technical difficulty of the procedure, which is not necessarily overcome after some training. Conversely, vibration and propagation of mucosa wave cannot be studied through any other more advanced, easy to access test as videokymography, making it a very promising test for research purposes and clinical practice.

## CONCLUSION

Based on the study performed with three female subjects without vocal complaints to check the correspondence of videokymography images with variation of laryngeal exposure in relation to the telescope, we concluded that there were evident differences in the obtained images, which requires standardization of laryngeal exposure for correct interpretation of videokymography.
